# Exploring Dimensionality Reduction Techniques for Deep Learning Driven QSAR Models of Mutagenicity

**DOI:** 10.3390/toxics11070572

**Published:** 2023-06-30

**Authors:** Alexander D. Kalian, Emilio Benfenati, Olivia J. Osborne, David Gott, Claire Potter, Jean-Lou C. M. Dorne, Miao Guo, Christer Hogstrand

**Affiliations:** 1 Department of Nutritional Sciences, King’s College London, Franklin-Wilkins Building, 150 Stamford St., London SE1 9NH, UK; alexander.kalian@kcl.ac.uk; 2 Istituto di Ricerche Farmacologiche Mario Negri IRCCS, Via Mario Negri 2, 20156 Milano, Italy; emilio.benfenati@marionegri.it; 3 Food Standards Agency, 70 Petty France, London SW1H 9EX, UK; olivia.osborne@food.gov.uk (O.J.O.); claire.potter@food.gov.uk (D.G.); david.gott@food.gov.uk (C.P.); 4 European Food Safety Authority (EFSA), Via Carlo Magno 1A, 43126 Parma, Italy; jean-lou.dorne@efsa.europa.eu; 5 Department of Engineering, King’s College London, Strand Campus, Strand, London WC2R 2LS, UK; 6 Department of Analytical, Environmental and Forensic Sciences, King’s College London, Franklin-Wilkins Building, 150 Stamford St., London SE1 9NH, UK

**Keywords:** QSAR, dimensionality reduction, deep learning, autoencoder, principal component analysis, locally linear embedding, grid search, hyperparameter optimisation, mutagenicity, cheminformatics

## Abstract

Dimensionality reduction techniques are crucial for enabling deep learning driven quantitative structure-activity relationship (QSAR) models to navigate higher dimensional toxicological spaces, however the use of specific techniques is often arbitrary and poorly explored. Six dimensionality techniques (both linear and non-linear) were hence applied to a higher dimensionality mutagenicity dataset and compared in their ability to power a simple deep learning driven QSAR model, following grid searches for optimal hyperparameter values. It was found that comparatively simpler linear techniques, such as principal component analysis (PCA), were sufficient for enabling optimal QSAR model performances, which indicated that the original dataset was at least approximately linearly separable (in accordance with Cover’s theorem). However certain non-linear techniques such as kernel PCA and autoencoders performed at closely comparable levels, while (especially in the case of autoencoders) being more widely applicable to potentially non-linearly separable datasets. Analysis of the chemical space, in terms of XLogP and molecular weight, uncovered that the vast majority of testing data occurred within the defined applicability domain, as well as that certain regions were measurably more problematic and antagonised performances. It was however indicated that certain dimensionality reduction techniques were able to facilitate uniquely beneficial navigations of the chemical space.

## 1. Introduction

Food and drink products contain a variety of chemicals, of which some are intentionally present (e.g., additives) while others are unintentionally present (e.g., environmental contaminants) [1]. The possible risks posed by consumption of food and drink chemicals to human and animal health, are ideally evaluated via chemical risk assessments (CRAs) carried out by regulatory bodies, industry and other stakeholders [1]. Current CRA frameworks are overly reliant on in-vivo studies on live animals [1], which pose significant ethical, scientific relevancy, and scalability-based concerns for the future of this space. New Approach Methodologies (NAMs), including *in-silico* approaches such as Quantitative Structure-Activity Relationship (QSAR) modelling, may be used to address some of these limitations and contribute to future frameworks that regulatory bodies may utilise [2].

QSAR modelling relies on the premise that molecular structures are correlated with corresponding biological activities [2]; hence, in a toxicological context, QSAR models quantify relationships between toxicological properties of molecules and quantitative metrics concerning molecular structures. There is no universally agreed upon metric for reliably quantifying molecular structures [2], however a variety of different approaches exist, such as structural similarity coefficients (SCs—which quantify structural similarities between pairs of molecules via techniques such as molecular fingerprinting [2]). QSAR models frequently use machine learning (ML) to construct models; in a toxicological context, this typically entails regression algorithms that can predict continuous metrics [3]. However, classification algorithms are more appropriate for certain endpoints that entail categorical data (rather than continuous numerical data) and are hence associated with discrete predictions [3]—e.g., mutagenicity is an endpoint where molecules may be assessed into discrete classes: A (strongly mutagenic), B (weakly mutagenic) and C (non-mutagenic) [4]. 

Mutagenicity concerns the ability of molecules to induce genetic mutations [4]; this endpoint is hence the subject of considerable research attention, especially due to its relevance to cancer biology. Additionally, mutagenicity is a widely covered endpoint in existing QSAR modelling literature, due to a relatively abundant pool of open-source data available, typically obtained via *in-vitro* Ames mutagenicity tests [4]. QSAR models of mutagenicity were compared as part of the 2014 Ames/QSAR International Challenge Project (2014 AQICP), involving a variety of rule-based QSAR frameworks such as SARpy and Toxtree, as well as statistical QSAR frameworks such as CEASAR and AMBIT. Sensitivity and specificity scores varied, however none were able to consistently surpass accuracy scores of 80% (considering both sensitivity and specificity), furthermore with many displaying imbalance across classes [4]. Deep neural networks (DNNs) are a popular ML technique, using layers of artificial neurons with weights that may be optimised to form complex models. A 2021 study used feed-forward DNNs to build a QSAR model of mutagenicity which was 84% accurate [5], while a separate 2021 study used graph convolutional networks (GCNs) in order to obtain sensitivity scores that were consistently matching or below 70% and specificity scores that were consistently above 90% [6]. 

Although DNNs have demonstrable advantages for QSAR modelling, naturally high dimensionality may arise in feature spaces built from chemical descriptors, hence deep learning based QSAR model performances may be impaired by “the curse of dimensionality”, where computational cost for a sufficiently complex model scales unfeasibly with increased dimensionality [7]. This may be alleviated via dimensionality reduction techniques, which either select or extract features to produce lower dimensional spaces, while aiming to conserve as much useful information as possible, to aid subsequent model simplicity and performance [7]. Our recent research used the 2014 AQICP dataset to construct feature vectors from SCs and fragment occurrences, for deep learning based QSAR models, with initial dimensionalities in excess of 10^4^ [8]. Principal component analysis (PCA) was used to reduce dimensionality to within the 10^2^ order of magnitude, which enabled overall accuracy scores of ~70% (for individual models using single types of feature vector) and ~78% (for combined models, using both types of feature vector) [8]. Although demonstrably effective, PCA is a linear dimensionality reduction technique that may fail to sufficiently conserve any information that exists across higher dimensional manifolds; the authors of the paper indeed acknowledged this limitation and noted that a potential expansion of their study could entail the exploration of alternative dimensionality reduction techniques, particularly non-linear techniques [8]. Other studies involving deep learning driven QSAR models have made use of dimensionality reduction techniques such as PCA, genetic algorithms, locally linear embedding (LLE), autoencoders and others, however the choice of specific algorithm has frequently been trivial and relatively few comparisons have been drawn, hence a research gap has been identified for specifically exploring optimisation and performance of dimensionality reduction algorithms in this space [7,8,9,10,11].

The aim of this study was therefore to explore, optimise and directly compare the performance of a diverse variety of dimensionality reduction techniques (both linear and non-linear) for enabling deep learning QSAR models of mutagenicity to navigate higher dimensional toxicological/chemical space as effectively as possible. 

## 2. Materials and Methods

### 2.1. Hypothesis and Research Method Overview

Cover’s theorem statistically shows that for any *N* binary labelled datapoints situated in a *D*-dimensional space, there is a high likelihood for the data to be linearly separable if *N ≤ D* + 1 (and this probability converges towards 1, as $\frac{D}{N}$*→∞*) [12]. While certain non-linearly separable configurations of high-dimensionality data are possible, the number of possible linearly separable configurations grows exponentially larger than the number of possible non-linearly separable configurations, as dimensionality *D* increases [12]. It is still possible that some high-dimensionality toxicological datasets may entail certain relationships between datapoints that result in non-linearly separable data, however this is unlikely from a statistical perspective [12]. It should also be considered that even a non-linearly separable dataset may be *approximately* linearly separable, with only a comparatively small number of datapoints mishandled by a linear decision boundary.

The total number *N* of datapoints to be used in this study will be equal to the dataset dimensionality *D*, however the number of training datapoints in each case will be lower (80% of the total balanced data, in each case). It is hence hypothesised that, in accordance to Cover’s theorem, linear dimensionality reduction techniques will be sufficient for enabling optimal performance of the deep learning driven QSAR models of mutagenicity trained and tested within this study, but that nonetheless certain non-linear dimensionality reduction techniques will perform equivalently well, while posing as more generally applicable techniques to other datasets where *N > D* + 1 or otherwise where non-linearly separable distributions may be anticipated to occur.

### 2.2. Data Collection and Pre-Processing

The 2014 AQICP dataset was used, following curation measures such as cross-referencing canonical SMILES (simplified molecular-input line-entry system) and CAS Registry Number descriptors via the online chemical database PubChem [13], as well as checking for sufficiently complete Ames mutagenicity data. Only molecules which passed these initial protocols were included in this study, which resulted in a final curated dataset of 11,268 molecules. A further characterisation of this curated dataset may be found in Appendix A. From these 11,268 curated molecules, canonical SMILES descriptors were standardised via the open-source MolVS Python package (using the default MolVS function for SMILES standardisation, which makes use of various background operations via the open-source Python cheminformatics package RDKit, such as removing explicit H atoms, applying normalisation rules, reionising acidic groups and more) [14,15]. It should be noted that all Python software developed in this study used Python 3. Following successful standardisation of canonical SMILES, it was necessary to address a significant imbalance between different classes in the dataset; molecules listed under mutagenicity classes A and B were severely outnumbered by molecules listed under class C. In line with other mutagenicity QSAR studies that used the same dataset, classes A and B were combined into a single “mutagenic” class, whereas class C was assigned as a “non-mutagenic” class [4]. While this measure improved the balance between classes, it did not fully alleviate the issue, with the number of non-mutagenic molecules remaining considerably greater (10,188 non-mutagenic molecules and 1,080 mutagenic molecules). This demonstrates the appropriate nature of using combined mutagenic class, to assist in alleviating the issue of severe data imbalance as far as possible (and to empower any classification models used with a sufficient number of datapoints for given classes, which was most intuitively achieved via a combined single mutagenic class, rather than two even more sparsely populated A and B classes against a significantly larger C class). Subsequently, perfect balance in the training data used was enforced via stratification of the dataset into balanced folds, for use in *k*-fold cross validation. *k*-fold cross validation was deemed to be a suitable technique for use in this study, given that it is widely applied in similar QSAR modelling studies, as well as that it may enable insight into the entirety of the sampled dataset, avoiding universal exclusion of chosen testing data from training the model [16]. 5 folds were constructed via random sampling of an equal number of molecules from each class (corresponding to the total number of mutagenic molecules, divided by 5). This resulted in all mutagenic molecules assigned to folds, but with a considerable number of remaining non-mutagenic molecules that were unassigned; these non-mutagenic molecules were instead assigned to a pool of permanent testing data for assisting with later validation of models. Over each iteration of training and testing a QSAR model, training data consisted of the combined contents of 4 non-selected folds (i.e., 80% of the balanced fold-assigned data, but just 15% of the total data), with the testing data composed of the selected fold (i.e., 20% of the balanced fold-assigned data) combined with the permanent pool of excess non-mutagenic testing data (hence in total, 85% of the total data assigned as testing data).

**Figure 1:**
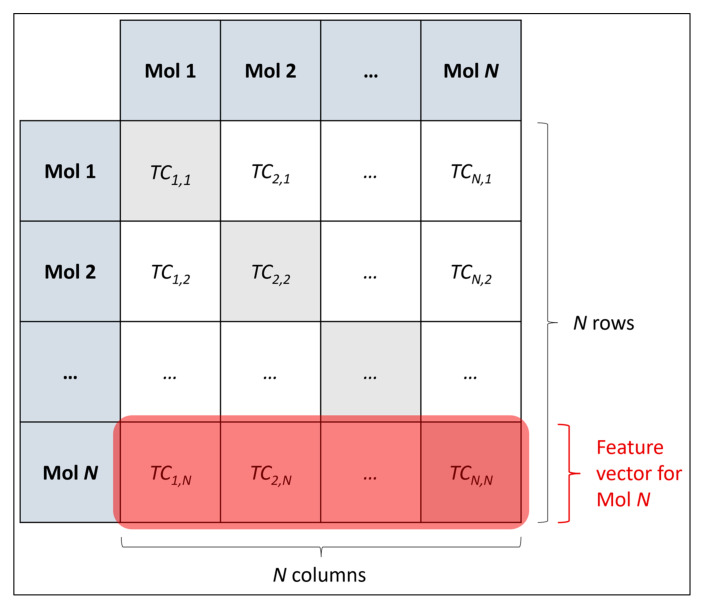
Schematic diagram of how an *N × N* matrix of TCs was constructed as a feature space for *N* molecules, along with corresponding feature vectors for each molecule. In this study, *N* = 11,268.

Feature engineering took place, for the purpose of adequately quantifying molecular structures, via a SC-based approach closely comparable to that used in our previous research [8]. A matrix of Tanimoto coefficients (TCs) was calculated, quantifying structural similarities between standardised canonical SMILES for each molecule in the curated dataset and every other molecule (this included trivial TCs between the same molecule, with perfect similarities of 1.0, for the purpose of maintaining an intuitive square matrix with consistency in the rows and columns of matrix corresponding to particular molecules) [8]. Functions from RDKit were used to compute TCs, upon first converting canonical SMILES to Morgan fingerprints (using a specified radius of 3 atoms and a maximum size of 2048 bits) [15,17]. The obtained matrix of TCs was the initial feature space, containing 126,967,824 (11,268^2^) TCs, with each row as a feature vector (each with a dimensionality of 11,268, to be later reduced) for each molecule as a sample; this may be visualised below in Figure 1:

### 2.3. Overview of Dimensionality Reduction Techniques

The dimensionality reduction techniques explored in this study were PCA, kernel PCA (kPCA), independent component analysis (ICA), autoencoders, LLE and isomap. PCA and the version of ICA used in this study are linear dimensionality reduction techniques, whereas kPCA, autoencoders, LLE and isomap are non-linear methods [18,19]. 

PCA is a statistical method that attempts to transform higher dimensional data into a lower dimensional space, according to explained variance along different orthogonal axes [20]. kPCA follows the same underlying principles as PCA, but makes use of a kernel for the purpose of more effectively transforming data that may not be linearly separable [19]. The kernel used may trivially be a linear kernel and hence equivalent to PCA, but more characteristically may be a non-linear kernel such as a radial basis function (RBF), sigmoid function or others. ICA attempts to separate data into separate components, via the assumption that those components are distributed in a statistically independent manner, with a number of more specific algorithms existing [21]. Autoencoders are a specific type of DNN with architectures (an encoder and decoder connected by a bottleneck) that naturally transform input data into lower dimensional encoded states, before decoding to the original state [10,22]. An autoencoder may hence be trained to encode and then decode data to a sufficiently accurate degree, upon which the encoder may then be used to perform dimensionality reduction on the data [10,22]. LLE is an algorithm which quantifies linear relationships between neighbouring datapoints (although these combined linear relationships may accurately represent a set of points occurring across a non-linear manifold), before then transforming the data into a lower dimensional space where such relationships are conserved as much as possible [11,18]. Isomap is comparable to LLE, aiming to conserve relationships between neighbouring datapoints that are transformed into a lower dimensional space; however, isomap quantifies geodesic relationships between neighbours and uses a different set of algorithms to compute the transformation [18,23].

All algorithms, with the exception of autoencoders, were implemented via available functions of the open-source Python ML library scikit-learn [24]. Autoencoders however were unavailable as a function of scikit-learn and hence were constructed as an object using the open-source Python ML library TensorFlow, with the encoder and decoder parts implemented as constituent objects [25].

### 2.4. Grid Search for Hyperparameter Optimisation

Before drawing a direct comparison between these dimensionality reduction techniques, it was first imperative to optimise their hyperparameters, to enable a fair and valid comparison [26]. While scikit-learn had numerous default values for the hyperparameters of each technique’s function, grid searches for each technique were performed over the top 2 hyperparameters deemed as most important [25,26]. The hyperparameters and their values were explored as follows:

- PCA, as a comparatively simpler technique and with already demonstrated effectiveness via default values in our previous research (which used the same dataset and closely comparable feature engineering), was neglected from grid search efforts; default values for more trivial hyperparameters were simply used [8,20,24].
- kPCA was similarly not considered for a grid search, although 2 different kernels were used for comparison purposes: an RBF function and a sigmoid function. Aside from this, default values for more trivial hyperparameters were used [24].
- The FastICA algorithm was used for ICA, with whitening strategy varied between use of arbitrary variance (default) and use of unit variance (note that it was assumed that the data was not already whitened), as well as maximum number of allowed iterations of the algorithm linearly varied between 200 (default) and 1000, with steps of size 100. Aside from this, default values were used for other hyperparameters [24].
- Autoencoders were configured using the sigmoid activation function in encoder neurons, whereas the ReLU activation function was used in the decoder neurons. The Adam optimisation algorithm was used for stochastic optimisation, along with mean squared error to compute losses. Shuffling of data between epochs was enabled, whereas the number of epochs was varied geometrically between 10 and 1280 (doubling with each step). The number of layers in the encoder and decoder were kept equal and varied via “number of steps to bottleneck” which referred to the number of layers before and after the bottleneck layer, for the encoder and decoder respectively; this number was varied linearly between 1 (for the most simple type of autoencoder with 3 layers in total) and 5 (a more complex autoencoder with 11 layers in total). For each given value for number of steps to bottleneck, the size of the first and last autoencoder layer would always be 11,268 (the original dimensionality), whereas the size of the bottleneck layer would be that of the chosen latent space, however the sizes of the layers between was configured as a geometric series—e.g., for the decoder layers to start from the latent space size and reach 11,268 within the chosen number of steps, maintaining the same common ratio each time. This common ratio naturally changed for different values for number of steps to bottleneck layer and hence was recalculated for these different values. Each common ratio was equivalently used for configuring the sizes of encoder layers, except via division rather than multiplication (as the geometric series would operate in reverse). Any decimal values for layer sizes obtained via these geometric series were simply rounded to the nearest whole number.
- LLE was varied in terms of strength of regularisation constant and number of neighbours considered for each point. The strength of the regularisation constant was varied geometrically between 10^−6^ and 10^−2^ (note that the default value was 10^−3^), with a common ratio of 10. The number of neighbours considered was varied linearly between 5 (default) and 115, with step sizes of 10. Aside from this, default values were used for other hyperparameters [24].
- Isomap was varied in terms of number of neighbours considered for each point, as well as eigenvalue decomposition algorithm. The number of neighbours considered was varied in an identical manner to that of LLE, whereas eigenvalue decomposition algorithm was varied between Arnoldi decomposition and the LAPACK (linear algebra package) solver [24]. It should be noted that for Arnoldi decomposition, it was further necessary to specify a maximum number of iterations, which was hence varied in an identical manner to that used for the ICA hyperparameter grid search [24]. Aside from this, default values were used for other hyperparameters [24].

In all cases, the algorithms (at each state of chosen hyperparameters) were fitted to training data (a matrix of all feature vectors from 4 of the 5 folds) and then used to transform testing data (a matrix feature vectors from the remaining fold and the permanent testing data pool). This was repeated, using a different fold for the testing data each time, giving a total of 5 iterations. The reduced dimensional space was controlled at 100 dimensions in all cases, for the purposes of the grid search stage, to enable a suitably simple and valid search for hyperparameter value combinations. It is further noted that a reduced dimensionality of 100 (achieved via PCA) was found to be sufficient in our previous research [8]. Following dimensionality reduction across each combination of hyperparameters for each algorithm, simple DNNs were constructed to train on and classify the new data, via multi-layer perceptron (MLP) classifiers with 2 hidden layers containing 500 artificial neurons each. These MLPs were implemented through functions of scikit-learn and used the ReLU activation function, along with Adam optimisation and a maximum number of 1000 epochs (while all other hyperparameters were kept at default values) [24]. Obtained sensitivity and specificity scores (regarding mutagenic predictions as positive, while non-mutagenic predictions as negative) were used to calculate overall accuracy, via finding their weighted average (according to prevalence of each respective class, in the testing data).

### 2.5. Final Comparison of Dimensionality Reduction Techniques

Adopting the optimal hyperparameter values from the grid search stage, dimensionality reduction techniques were compared. Final dimensionality was varied over the following values: 2, 4, 8, 10, 20, 50, 100, 200, 300. These values were chosen to cover a sufficiently wide search space that would be likely to demonstrate convergence in performance, to a sufficiently large reduced dimensionality for conserving information. Upon obtainment of transformed feature spaces over all algorithms, iterations and dimensionalities, comparison was enabled via training and testing of identical MLP classifiers as described in Section 2.3. Metrics used for comparison included overall accuracy, sensitivity, specificity, positive predictive value (PPV) and negative predictive value (NPV).

### 2.6. Defining the Applicability Domain

There are numerous means for defining the applicability domain (AD), to characterise the applicability of QSAR models [27]. In this study, the AD was defined via first plotting XLogP (a computational approximation of LogP, i.e., partition coefficient) against approximate molecular weight (MW), for each molecule (with both metrics obtained from PubChem) [13]. The AD was then defined as a rectangular region of this two-dimensional chemical space, with boundaries defined by the minimum and maximum XLogP and MW values of the training dataset, for each *k*-fold iteration. Any molecules from the testing dataset with *(XLogP, MW)* coordinates outside of this region, were deemed as existing outside the AD.

## 3. Results and Discussion

### 3.1. Grid Search

**Figure 2:**
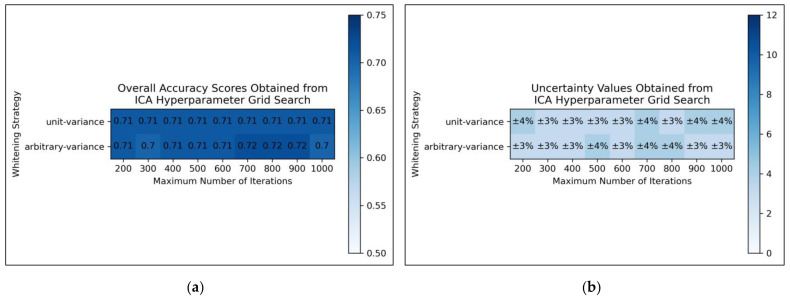
For ICA: (**a**) Heat map of mean overall accuracy scores obtained from grid search, over all 5 iterations; (**b**) Complementary heat map of percentage uncertainty values (standard deviation) on mean overall accuracy scores.

From the results of the grid search, it was deemed that for ICA, arbitrary variance performed marginally better than unit variance in terms of overall accuracy scores, with the most optimal scores occurring between 700 and 900 maximum iterations, although this was possibly a mere statistical fluctuation in otherwise considerably uniform data (Figure 2a). Uncertainty values appeared uniform and trivially fluctuating, although use of arbitrary variance overall gave rise to lower uncertainties than use of unit variance (Figure 2b). It was decided from this grid search that the ICA algorithm would use arbitrary variance and a maximum number of 800 iterations (a natural middle ground between 700 and 900).

**Figure 3:**
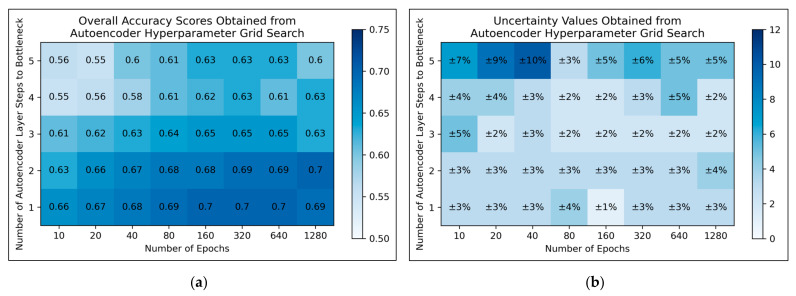
For autoencoders: (**a**) Heat map of mean overall accuracy scores obtained from grid search, over all 5 iterations; (**b**) Complementary heat map of percentage uncertainty values (standard deviation) on mean overall accuracy scores.

For autoencoders, a tendency for improved model performance was observed as hyperparameter states progressed to the lower-right corner of the heat map, i.e., minimal autoencoder complexity and larger numbers of epochs were optimal (Figure 3a). Affirming this, an approximate trend of lower uncertainty values was apparent along this direction in hyperparameter space (Figure 3b). Nonetheless, a slight decline in overall accuracy was present when increasing the number of epochs from 640 to 1280, for the simplest autoencoder architecture; the most optimal performances instead occurred between 160-640 epochs. Thereby, 320 epochs were selected for further use, as a natural middle ground of this optimal range, whereas the selected architecture would follow just 1 step to the bottleneck (i.e., an autoencoder with 3 layers in total).

**Figure 4:**
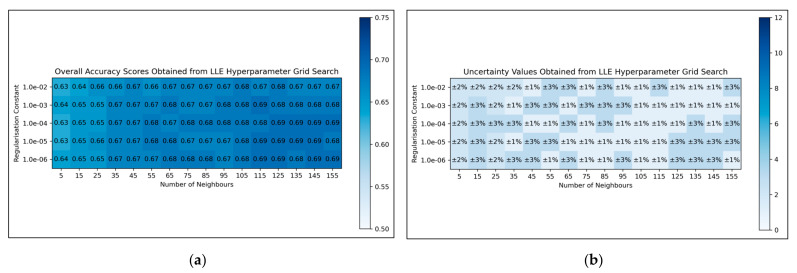
For LLE: (**a**) Heat map of mean overall accuracy scores obtained from grid search, over all 5 iterations; (**b**) Complementary heat map of percentage uncertainty values (standard deviation) on mean overall accuracy scores.

For LLE, a general trend was uncovered, of more optimal performances for increased numbers of neighbours and decreased regularisation constants (Figure 4a). This was less clearly reflected in uncertainty values, which appeared overall more uniform and to have otherwise varied in a random manner (Figure 4b). Although the overall trend in LLE performance is clear, it does not perfectly hold for every hyperparameter state. It was decided from these results that the regularisation constant would be controlled at 10^−6^, whereas the number of neighbours would be controlled at 115 (as this was the lowest number of neighbours, with the lowest uncertainty value, which resulted in the most optimal performance when using a regularisation constant of 10^−6^).

**Figure 5:**
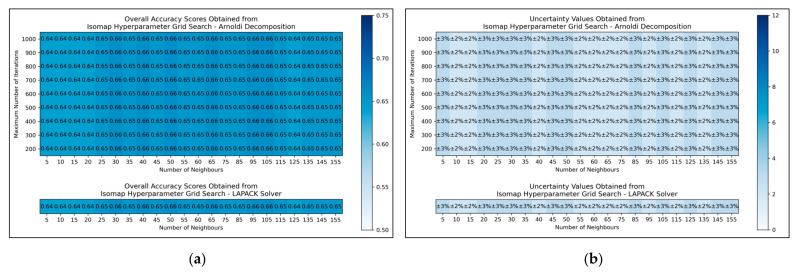
For isomap: (**a**) Heat map of mean overall accuracy scores obtained from grid search, over all 5 iterations; (**b**) Complementary heat map of percentage uncertainty values (standard deviation) on mean overall accuracy scores.

The results for the grid search for isomap shows negligible variation across varied numbers of neighbours considered, albeit with a slight improvement in average performance as numbers were increased beyond 20 (Figure 5a,b). No variation in performance or corresponding uncertainty occurred across different eigenvalue decomposition algorithms, nor for different maximum iteration values when using Arnoldi decomposition. Although the success of the isomap parameter optimisation was limited, the available evidence suggested that the number of neighbours would be optimally configured at 65. This configuration not only lead to the tied most optimal performance, along with lowest uncertainty, but also served as a natural middle ground between other values that performed comparably well. The eigenvalue decomposition algorithm was set as “auto” to enable the software to choose the most efficient algorithm of the two (both appeared to be identical in performance, through the grid search).

Overall, the grid search stage of this study was of mixed success. Clear and coherent optimisations were reached for autoencoders (Figure 3) and LLE (Figure 4), demonstrable via their heatmaps, although their optimal states were notably within proximity of the lower-left bounds of the heat maps. Hence a more optimal state may exist outside the bounds of the search space, which could be explored in future research. A less distinct optimised state was found for ICA (Figure 2), which may indicate that the search space was not wide or precise enough, or that alternatively other hyperparameters should have been varied instead. Despite this, some level of optimisation was still possible via the ICA grid search. The least success from the grid search however occurred for isomap (Figure 5), where the varied eigenvalue decomposition algorithms and maximum number of iterations (when using Arnoldi decomposition) had no observed impact on QSAR model performance. This indicates that the choice of including these hyperparameters for the isomap grid search was not optimal. While the search space for numbers of neighbours did have some level of variance in overall accuracy (Figure 5a), no clear trend was discernable and the variance was furthermore not clearly distinguishable from possible naturally occurring statistical noise in the data. This indicates that the search space for the number of neighbours hyperparameter was insufficient, even if it was sufficient for the LLE grid search (Figure 4).

Overall, grid search offers an appropriate approach for this study, as it would not have been feasible to choose hyperparameters, search spaces and required levels of resolution through any exact theoretical *a priori* deduction, for such a complex dataset containing 11,268 dimensions; instead a level of starting assumption was necessary. Nonetheless, a potential future research direction could be to expand considered hyperparameters, their respective search spaces and respective levels of resolution, based on the outcomes of an initial grid search (particularly based on whether sufficient optimisations could be found from the initial grid search). The uneven and arbitrary nature of the resolutions between different grid searches could also be more coherently standardised via a clear methodology, for a future study. Additionally, the inherent limitations of grid searches as a hyperparameter optimisation method (particularly the limitation of resolution of the search space and potentially missing more optimal states between chosen search values) could be addressed via choosing another optimisation approach such as random searches and Bayesian optimisation, perhaps to be ran in parallel to grid searches, followed by a final comparison of optimised hyperparameters found [26].

The grid search stage of the study provides an early level of insight into the feasibility of the explored dimensionality reduction algorithms and their respective chosen hyperparameters, as overall accuracy scores approximately between 60–70% were obtained, with comparatively insignificant levels of uncertainty. This indicates that all configured dimensionality reduction techniques were able to reduce the dimensionality of the feature space, while conserving sufficient information to empower simple MLP classifiers to perform measurably more accurately than a random guess and comparable to performance metrics obtained through our previous research [8]. While the controlled dimensionality of 100 may have inhibited certain techniques from producing more optimal feature spaces, the later stage of subsequent comparison of performances over numerous ascending dimensionalities would help alleviate this limitation, while the controlled value of 100 dimensions is deemed as having maintained an appropriate and feasible set of conditions for a valid prior grid search.

### 3.2. Comparative Performances of Dimensionality Reduction Techniques

**Figure 6:**
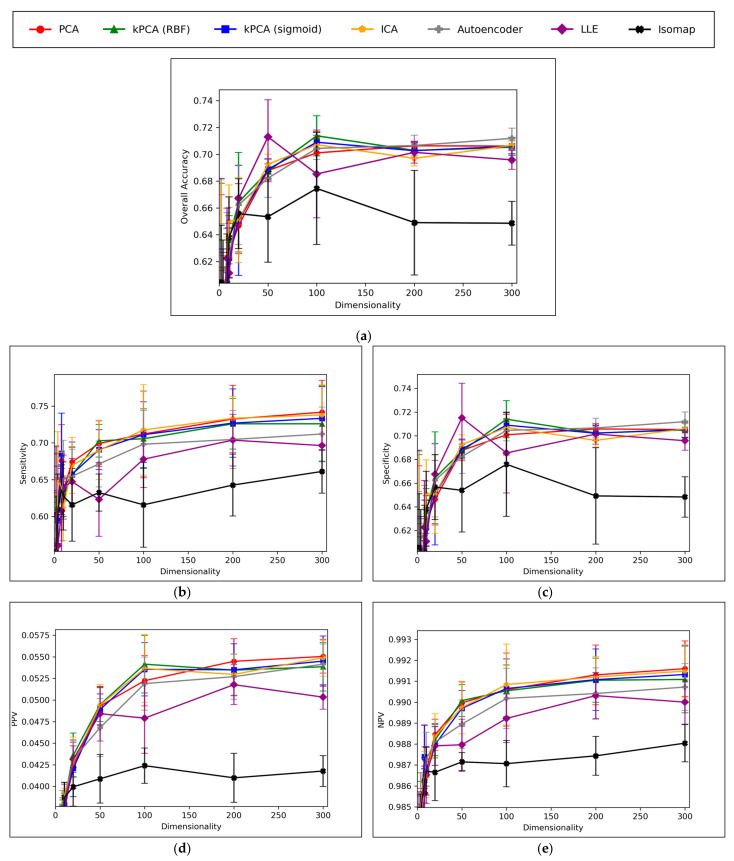
Comparative performance metrics graphs for all optimised dimensionality reduction techniques, over ascending dimensionalities, in terms of: (**a**) Overall accuracy; (**b**) Sensitivity; (**c**) Specificity; (**d**) PPV (positive predictive value); (**e**) NPV (negative predictive value); (Note that for aiding clarity in comparing more pertinent high performance results at higher dimensionalities, *y*-axis cutoffs exclude lower performances at lower dimensionalities.

Direct comparison of dimensionality reduction technique performances, using optimised hyperparameters, is illustrated in Figure 6:

A convergence of overall accuracy scores was observed, for the majority of models, at 100 dimensions and beyond (Figure 6a). However, the sensitivity scores showed a gradually increasing trend for the highest performing models, even beyond 100 dimensions (Figure 6b). For specificity scores, similar trends and findings were found to theose of overall accuracy scores, due to the imbalanced nature of classes in the data (Figure 6c). Furthermore, similar trends were present for PPV and NPV values, however the PPV values were significantly smaller than the NPV values, naturally due to the data imbalance between mutagenic and non-mutagenic molecules (Figure 6d,e).

The overall convergence displayed in Figure 6 (especially Figure 6a,c) suggested that optimal dimensionality was approximately reached via the dimensionality reduction techniques and hence no further useful information could be provided through increased dimensionality. This also implies that the search space and resolution of dimensionalities was sufficient. Although sensitivity scores in Figure 6b do not show an absolute convergence, the gradient of improvement for dimensionalities increased beyond 100 is notably more gradual and hence convergence is approximately inferable. Additionally, the imbalance of mutagenic molecules (significantly outnumbered by non-mutagenic molecules) may call into question the exact accuracy of any trends discerned solely in terms of sensitivity. 

Overall, the results indicate that accuracy scores peaked within range of ~70%, even for the best performing dimensionality reduction techniques, which is also in line with the results of our previous study [8]. It is however possible that autoencoders may have been capable of reaching higher peak overall accuracy scores, at higher dimensionalities, as evidenced by a less visibly converged gradient of improvement, as well as reaching a peak above all other techniques at the maximum tested dimensionality of 300. The best performing technique is not absolute or conclusive from the results, as PCA, both forms of kPCA, ICA and autoencoders performed with closely comparable average overall accuracies, furthermore with considerable overlap between margins of error. At certain dimensionalities, LLE also performed at comparable levels. PCA, both forms of kPCA and ICA demonstrated more robust sensitivity scores than any of the other methods, however more equivalent performance was observed from autoencoders and LLE in terms of specificity. 

The consistently high performance of linear dimensionality reduction techniques, often greater than the performance of non-linear techniques, strongly suggests that the original 11,268-dimensional dataset was linearly separable, or at least approximately so. This is in line with Cover’s theorem and the underlying hypothesis of this study [12]. Although non-linear techniques such as kPCA, autoencoders and LLE overall performed comparably to PCA and ICA (which further affirms the hypothesis), PCA and ICA as linear techniques offer generally simpler and often more efficient techniques for processing high-dimensionality datasets into more manageable feature spaces for deep learning driven QSAR models. That said, molecules in toxicological space are not randomly distributed and hence potential non-linearly separable relationships in higher dimensional toxicological datasets may be more appropriately handled with autoencoders and LLE, in the absence of any *a priori* knowledge, as they in any case can perform in a comparable manner to linear techniques for data that are linearly separable. It is hence a more robust strategy to use generally applicable non-linear dimensionality reduction techniques, such as autoencoders, not just for datasets where *N > D* + 1 (and hence with increased likelihood of non-linear separability), but also when *a priori* knowledge of the dataset’s separability is lacking and/or difficult to characterize.

Isomap significantly underperformed throughout the different graphs of Figure 6, in comparison to the other dimensionality reduction techniques explored. This however could be expected, given the failure of the isomap hyperparameter grid search to uncover any clear optimal state; isomap has demonstrated effectiveness in numerous other QSAR modelling studies and may have performed measurably better, given a more robust hyperparameter Optimisation and resulting final hyperparameter configuration. On this note, other dimensionality reduction techniques may have also been hindered by the limitations of the grid search stage of this study, hence while some level of conclusions may be drawn from the results of this study, it is also important to note the limitations of these findings. Similarly, the simplicity of the MLP classifiers with only 2 hidden layers of 500 neurons, may have capped the potential of the results to reach higher peak accuracy scores, although the controlled nature of the MLP classifiers nonetheless serves as a valid means for drawing comparison between competing dimensionality reduction techniques. Nonetheless, it is possible that the peak overall accuracy scores within range of ~70% from Figure 6a may have been due to the limitations of the MLP classifier stage of the QSAR model, rather than any of the employed dimensionality reduction techniques, hence a future expansion to the study may benefit from varying the complexity of the DNNs used for classification.

It was deemed that the chosen selection of PCA, kPCA (using two different non-linear kernels), ICA, autoencoders, LLE and isomap, was sufficiently diverse in providing a valid basis for drawing a limited comparison between different dimensionality reduction techniques, especially in terms of linear techniques vs non-linear, for their suitability in building deep learning based QSAR models of mutagenicity and other toxicological endpoints. However 6 dimensionality reduction techniques compared in this study is not an absolute representation of all dimensionality reduction techniques, especially with regards to the diverse space of non-linear methods; wider comparison could be explored in future research.

While it was intended that while all findings of this study would naturally be directly applicable to the specific dataset used, these results were also aimed to provide insight into the general suitability of the algorithms explored, for similar QSAR modelling applications in future. Nonetheless it may be appropriate to extend future studies to include additional datasets.

### 3.3. Analysis of Applicability Domain

The AD was calculated across all iterations and is irrespective of dimensionality reduction technique used. 

**Figure 7:**
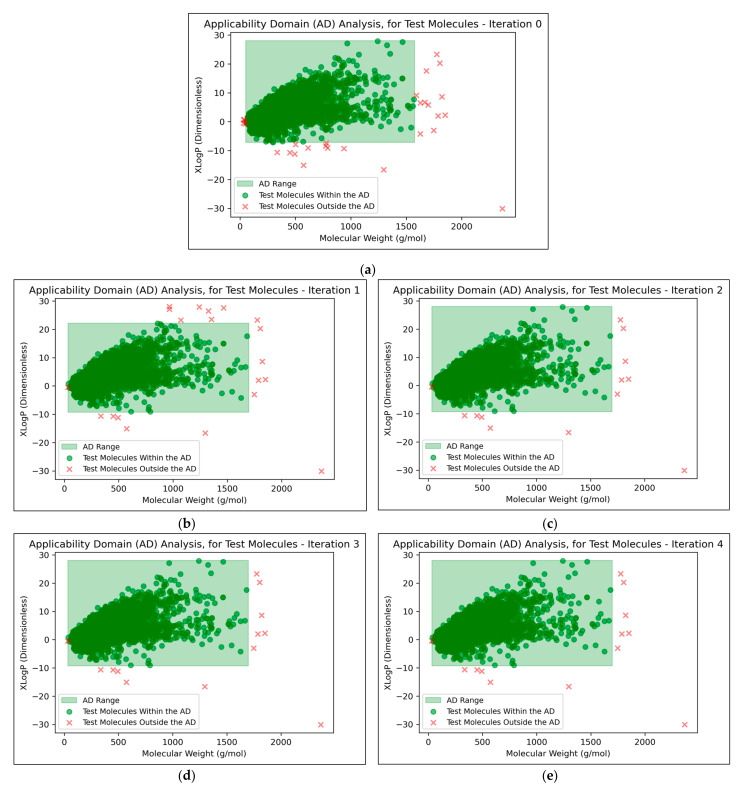
Visualisations of the AD and the positions of testing data molecules relative to it, for: (**a**) Iteration 0 (i.e., using fold no. 1 as testing data); (**b**) Iteration 1 (i.e., using fold no. 2 as testing data); (**c**) Iteration 2 (i.e., using fold no. 3 as testing data); (**d**) Iteration 3 (i.e., using fold no. 4 as testing data); (**e**) Iteration 4 (i.e., using fold no. 5 as testing data); Note that in all cases, total testing data was composed of the given test fold combined with a separate pool of permanently assigned testing data.

It was found that an average of 0.15% (2 sf, over all iterations) of molecules were located outside the AD (Figure 7). This comparatively low level indicated that the QSAR models used data that were sufficiently spaced and stratified, although it may additionally suggest that the definition of the AD used was limited and insufficient for explaining the ~30% of testing data that were misclassified at peak QSAR model performances (see Figure 6). It may also be observed from Figure 7 that some of the more prominent and/or eccentric datapoints outside of the AD are repeated in the testing data across different iterations; these datapoints hence represent non-mutagenic molecules that were assigned to the permanent testing data pool (and comparable outliers did not occur in the training data, as these would have widened the boundaries of the AD accordingly).

**Figure 8:**
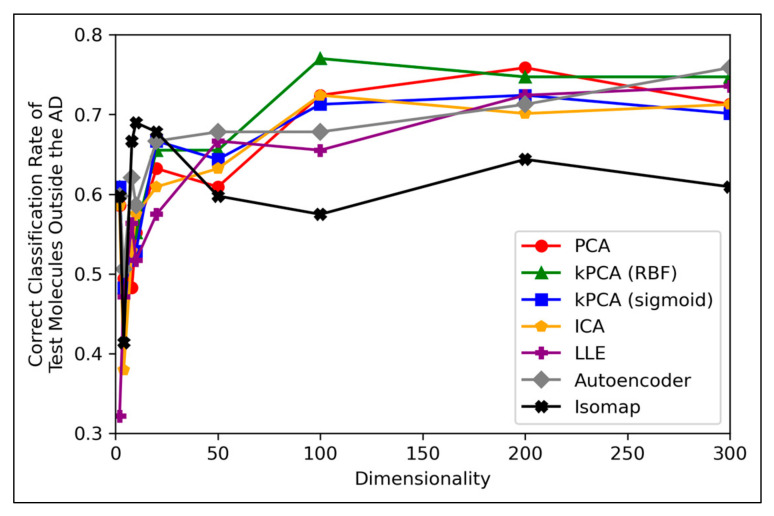
Line graph displaying correct classification rate (equivalent to overall accuracy) of molecules outside the AD for each given iteration, by QSAR models, over different reduced dimensionalities (and powered by different dimensionality reduction techniques).

It is generally expected that QSAR models are at risk of underperforming when used to test molecular data that exists outside of their AD, hence the performances of the QSAR models of this study, for classifying testing molecules outside of the AD, were investigated (Figure 8):

Figure 8 displays peak performances that were notably higher than the ~70% overall accuracy scores observed in Figure 6a, for all dimensionality reduction techniques except for isomap (although even isomap approached ~70% correct classification rate, at lower dimensionalities). Closely comparable trends were further found to hold in terms of specificity, however analysis in terms of sensitivity scores was limited, due to a scarcity of mutagenic molecules outside of the AD (see Appendix B). These findings are unexpected, as it demonstrates that the majority of QSAR models on average performed more optimally at classifying molecules outside the AD than at classifying molecules inside the AD. This could be due to the adopted definition of the AD for this study being insufficient. Although this is difficult to verify, as any conclusions drawn from comparisons between Figure 6 and Figure 8 may be inherently limited, due to the comparatively much lower amount of molecular datapoints that were available for constructing Figure 8 (0.15% of molecules, compared to 100% of molecules used to construct Figure 6). Nonetheless, another interesting finding from Figure 8 is that while techniques such as PCA, kPCA and ICA remained largely stagnant in performance at 100 dimensions and beyond, autoencoders and LLE continued to gradually improve in classification rate (with autoencoders outperforming all other techniques at 300 dimensions). This may indicate that non-linear dimensionality reduction techniques, particularly autoencoders, at a larger number of dimensions, could perform more optimally than linear techniques for processing data that contains a larger number of statistical outliers. The fact that a similar indication was also present from Figure 6a is reaffirming of the possibility that autoencoders may have reached more optimal overall accuracy scores at higher dimensionalities. It should however be noted that PCA, kPCA and ICA outcompeted autoencoders and LLE at lower dimensionalities of 100, despite PCA and ICA being linear techniques, as well as that PCA and kPCA underwent no grid search hyperparameter optimisation stage. kPCA, when using the RBF function as a kernel, surpassed 75% correct classification rate at 100 dimensions and was the most consistent top-performing technique in Figure 8. Similar findings for kPCA (RBF) at 100 dimensions are also observable from Figure 6a. The frequently more optimal performance of kPCA (RBF), compared to PCA and even kPCA (sigmoid), suggests that a RBF kernel was indeed more optimal for navigating the initial feature space.

Considering the above findings, it was deemed beneficial to obtain further insight into the classes of molecules which were outside the AD, as well as generally how the classes of molecules were distributed according to the chemical space used to the define the AD.

**Figure 9:**
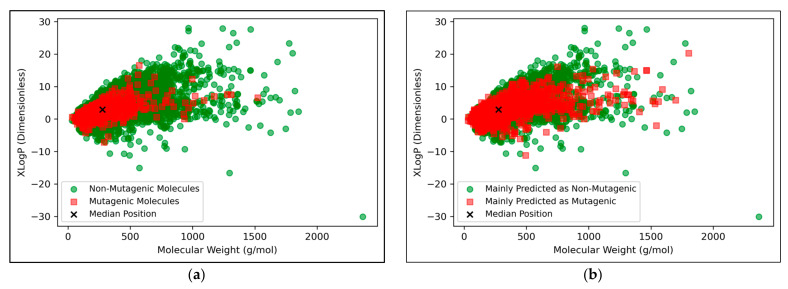
Visualisations of the distributions in chemical space of: (**a**) Molecules in the dataset that were mutagenic versus non-mutagenic; (**b**) Molecules in the dataset that were mainly predicted as mutagenic versus mainly predicted as non-mutagenic (according to the autoencoder powered QSAR model at 300 dimensions, across all iterations); Note that in all cases, median position of the entire dataset is marked, for reference. For an alternative colour-scheme, see Appendix C.

The vast majority of molecules outside the AD were non-mutagenic, along with a general majority of statistical outliers from the main cluster body of the visualised chemical space (Figure 9a). This may be a purely probabilistic phenomenon, as a significantly larger number of non-mutagenic molecules occurred in the curated dataset, compared to mutagenic molecules, which is supported by the observation that a minority of mutagenic molecules also appear as visible outliers, to a certain extent. This suggested that a minority percentage of both classes naturally were outliers in terms of their distribution in XLogP/MW chemical space, but that non-mutagenic cases were simply more frequent due to the inherent data imbalance. Regardless of this, the significance of the findings of Figure 8 may be called into question, given the extremity of the data imbalance of the molecules outside the AD, implying that the QSAR models were *de-facto* only being compared by their ability to classify non-mutagenic outliers; their overall improved performances may suggest that the MLP classifier simply learned to classify the majority of statistical outliers as non-mutagenic (an explanation which is supported by Figure 9a). While initial stratification of the training data attempted to remove any bias occurring in favour of any particular class, inherent limitations of the distribution of the classes in chemical space would have been less easily overcome and appears as a possible fundamental limitation of the 2014 AQICP dataset. Additionally, the success of the QSAR models in identifying statistical outliers of the physicochemical XLogP/MW chemical space as non-mutagenic, is an affirmation of the suitability of the techniques used. This is because the QSAR models demonstrated predictions that were directly interpretable via the physicochemical space used for defining the AD, despite having used feature vectors that were more abstract quantifications of toxicological space, via matrices of TCs between SMILES descriptors.

**Figure 10:**
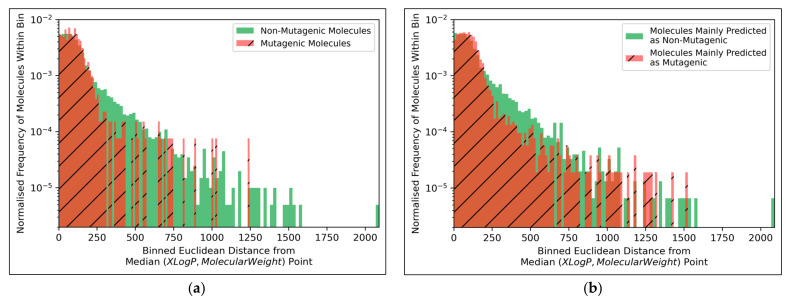
Histograms of Euclidean distances from the median point in XLogP/MW chemical space for: (**a**) Molecules in the dataset that were mutagenic versus non-mutagenic; (**b**) Molecules in the dataset that were mainly predicted as mutagenic versus mainly predicted as non-mutagenic (according to the autoencoder powered QSAR model at 300 dimensions, across all iterations); Note that 1000 bins were used for producing both histograms.

Figure 9b appears to display a considerably wider or less dense distribution of mutagenic predictions than that of the true distribution of Figure 9a, but the large density of datapoints makes this difficult to compare. Normalised histograms of distances from the median point were hence plotted in Figure 10.

The distributions of both classes of molecules, in terms of distance from the median point in XLogP/MW chemical space, were exponential distributions and approximately equivalent, except for the greater definition of the non-mutagenic exponential distribution, especially across further distance bins, owing to the inherent data imbalance (Figure 10a). This points to the main limitation of the 2014 AQICP dataset as concerning the inherent data imbalance, rather than any unequal distribution of the classes. The results also demonstrated that the MLP classifier mostly followed a balanced prediction of mutagenic and non-mutagenic molecules, over the combined distribution, with the exception of extreme outliers (Figure 10b). Certain characteristics of each distribution (such as the lower normalized frequencies of mutagenic molecules, compared to non-mutagenic molecules, spanning over bins ~200 to ~500) were captured by the MLP classifier. While this may indicate sufficient ability of the simple MLP classifiers for capturing subtle differences between distributions of mutagenic and non-mutagenic molecules in chemical space, it may also be a sign of overfitting.

**Figure 11:**
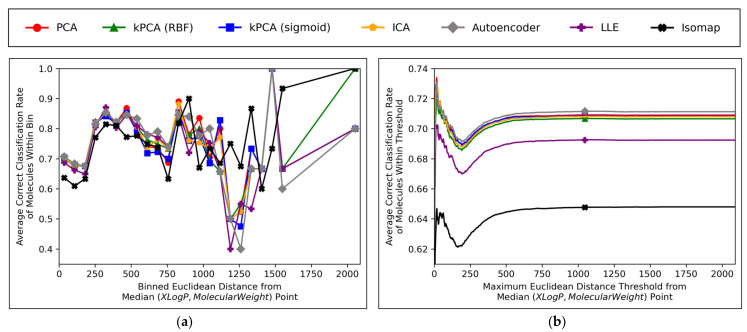
Graphical plots showcasing: (**a**) Average correct classification rate for molecules, over binned Euclidean distances from the median point in XLogP/MW chemical space (note that 30 bins were used and that some spaces between plotted points are uneven, due to exclusion of empty bins); (**b**) Average correct classification rate for molecules within maximum thresholds for Euclidean distances from the median point in XLogP/MW chemical space (note that 1000 thresholds were used); Also note that both graphs are concerning QSAR models that used 300-dimensional data, over all iterations.

Further insight into the performances of the different dimensionality reduction techniques, over differing Euclidean distances from the median point in XLogP/MW chemical space, were obtained via the graphical plots of Figure 11.

A universal dip in performance occurred, for molecules closest to the median point in XLogP/MW chemical space (Figure 11a), although it is suggested that this dip occurred later (Figure 11b) and hence the earlier positioning of the dip in Figure 11a appears to have been due to the imprecise nature of the 30 bins used. An additional dip in performance is visible in Figure 11a, having occurred for all techniques except for isomap, between *x*-axis values of ~1100 and ~1300. While this additional dip was also apparent in the raw data used to construct Figure 11b, the thresholded nature of Figure 11b (where averages are drawn over the entire dataset *within* a certain distance) means that higher average values were maintained from a denser pool of higher-performance molecules at closer Euclidean distances, with negligible impact from any lower performances over less densely populated farther regions. Isomap generally performed with local correct classification rates of 70–80% (Figure 11a), but this figure was lowered by initially poorer performance over the significantly more densely populated region of chemical space surrounding the median point. This nonetheless may suggest that isomap, despite having underperformed in terms of all other measures in this study and having seemingly not undergone a successful grid search for hyperparameter optimisation, could possibly offer unique advantages in coverage of sparser regions of chemical space. This would further suggest that perhaps the hyperparameter optimisation was more successful than previously realised, but that merely the performance metrics used for comparison were inherently flawed and biased towards favouring other algorithms which naturally performed more optimally over the densest central region of the XLogP/MW chemical space.

The initial performance dip region of the chemical space is most coherently displayed by Figure 11b, which affected all QSAR models to a closely comparable extent, regardless of dimensionality reduction technique used. This suggests that the particular region of chemical space in question was fundamentally problematic. Overall accuracy score shown in Figure 11 was significantly hindered by this performance dip over a considerably more dense region of the chemical space (see Figure 10); overall accuracy scores more within 80% may have otherwise been achieved, as per the more successful QSAR model performances observed over other regions in Figure 11a. It is nonetheless unclear why such a dip occurred over a distinctly problematic region, with such extensive data availability, nor how the region is spatially distributed (it is so far only characterised by a directionless distance range from the median point, but may be concentrated in a more specific location of an XLogP/MW plot). A future expansion of this study may hence entail a deeper investigation into more problematic regions of the considered chemical space, as well as potentially exploring models that can identify and exclude (or otherwise assign lower reported confidence levels on predictions from) molecules within such regions.

Aside from the above observations, Figure 11b demonstrates that the performances of PCA, kPCA (both types of kernel), ICA and autoencoders were closely comparable, which further affirms the hypothesis that non-linear dimensionality reduction techniques would be sufficient for navigating *N ≤ D* + 1 training data (as per Cover’s theorem) [12], but that certain non-linear techniques would perform sufficiently too (while inherently being more widely applicable to any non-linearly separable datasets used in future QSAR modelling applications). Although autoencoders mildly outperformed all other techniques in Figure 11b, this is deemed as having been due to the use of results arising from 300-dimensional data, which (as per Figure 6a and Figure 8) represents a particular point where autoencoders transiently outperformed other techniques; 300 dimensions was merely chosen as an arbitrary control, where some extent of valid further analysis over the XLogP/MW chemical space, as used for defining the AD, would be possible.

**Figure 12:**
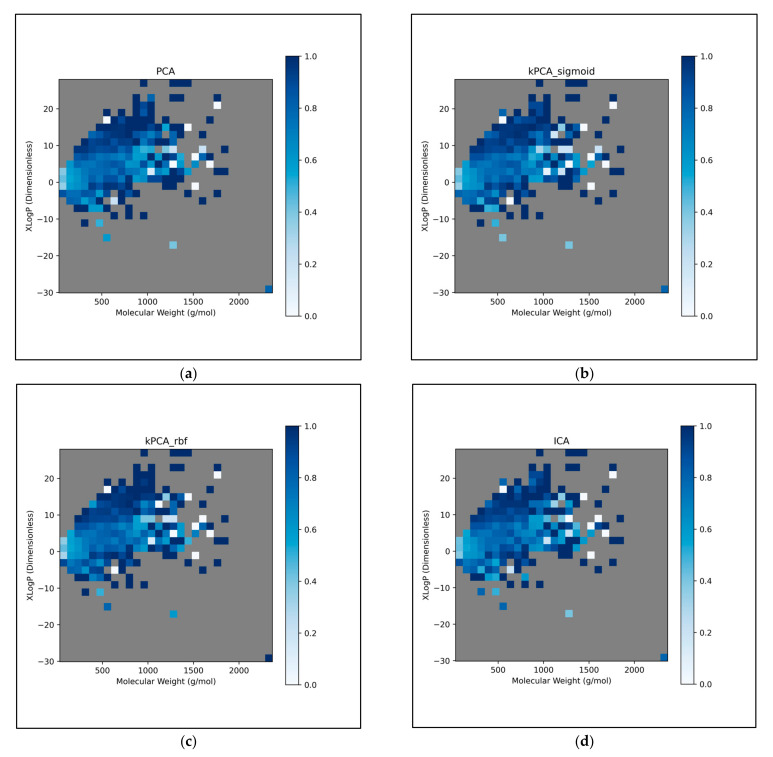

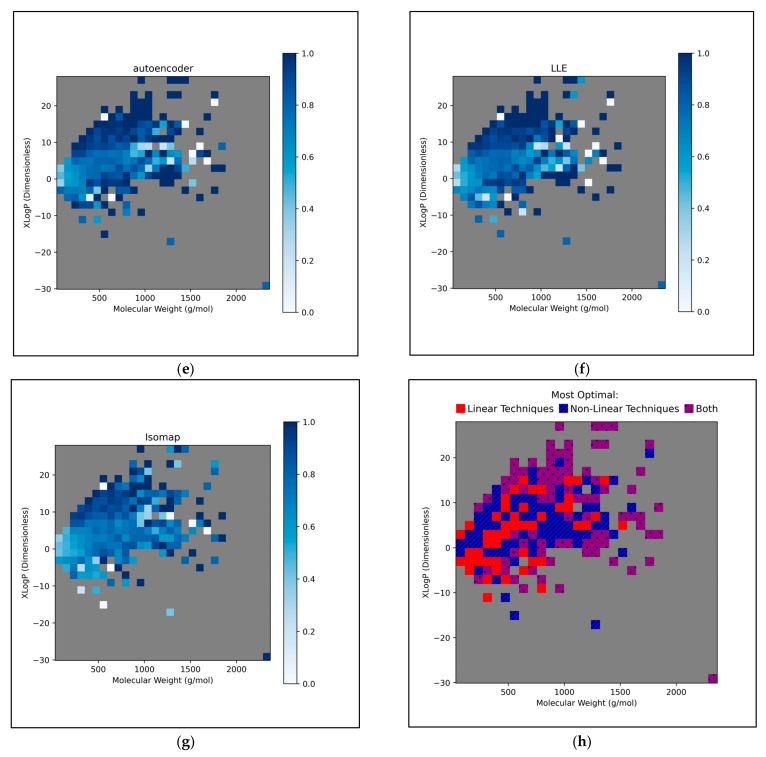
Heat maps of chemical space region-specific overall QSAR model performance at 300 dimensions, over all iterations, for: (**a**) PCA; (**b**) kPCA (sigmoid function); (**c**) kPCA (RBF); (**d**) ICA; (**e**) Autoencoders; (**f**) LLE; (**g**) Isomap; A further plot (**h**) displays whether linear (red with no lines) or non-linear (blue with diagonal lines) techniques or both equally (purple with dotted pattern) performed most optimally, for the given regions; Note that in all cases, chemical space was discretised into 30 equal bins of MW, along with 30 equal bins of XLogP, with empty bins of no data coloured grey.

The trends of Figure 11 were further explored, by directly analysing performances over different discretised regions of the XLogP/MW chemical space:

This further exploration of QSAR model performance, over all chemical space, uncovered compatible trends with those of Figure 11 (Figure 12). For all dimensionality reduction techniques, a certain region, within the densely populated region the median point, underperformed compared to similarly close and dense regions (Figure 12a–g). This region occurred directly to the left of the median point and appears to be responsible for the universal dips in performance observed in Figure 11b. In terms of differences in performances over different regions, between different dimensionality reduction techniques, distinct differences were found that were supportive of Figure 11a. Isomap in particular was found to have performed more uniformly than other techniques, albeit in most cases at a lower overall accuracy, however this held advantages over certain regions of chemical space (mostly to the right of the median point) that were problematic or of mixed performance, for other techniques. Although the idea that each technique could offer a unique insight into toxicological space was demonstrated to a certain extent, many wider trends concerning problematic regions were found to be universal. Further investigation revealed an overall balance in superiority of linear and non-linear techniques, over the studied discretised regions of the chemical space (Figure 12h).

## 4. Conclusions

Our extensive results and analyses presented in this study concluded that the original hypothesis was largely affirmed; although higher dimensional toxicological space may contain complex relationships that require conservation via dimensionality reduction, the statistical argument of Cover’s theorem appears to hold for this application [12]. The 11,268-dimensional training data, containing *N ≤* 11,268 datapoints, behaved as at least approximately linearly separable, as it was sufficiently navigated via linear dimensionality reduction techniques such as PCA and ICA, while non-linear techniques failed to outperform in any significant way (although this may not necessarily hold for datasets of other endpoints or even for other mutagenicity datasets). The hypothesis however was further affirmed in its prediction that certain non-linear techniques would demonstrate comparably optimal performance to linear techniques, while naturally holding the advantage of being capable of wider applicability to non-linearly separable datasets; this was indeed the case for kPCA, autoencoders and (to a somewhat lesser extent) LLE. Although kPCA frequently outperformed autoencoders in terms of final QSAR model performance, autoencoders displayed some indication of potential to outperform for higher reduced dimensionalities, while also carrying the advantage of not being limited through a pre-specified kernel function that kPCA requires *a priori* [19]. 

The grid search for hyperparameter optimisation was of mixed success, especially performing poorly for isomap, while none of the optimised dimensionality reduction techniques were able to consistently or significantly outperform PCA or kPCA (of which both did not undergo any optimisation process). The arbitrary assumptions used for performing the grid search, over limited sets of hyperparameters, as well as limited search spaces and resolutions, were identified as key weaknesses to be addressed in future studies, although the grid search used in this study may be regarded as having successfully enabled some extent of fair and valid comparison of advanced dimensionality reduction techniques, despite its key limitations. 

The vast majority of molecules tested occurred within the defined AD, although further analysis cast mixed certainty over the suitability of the possibly oversimplistic AD definition used. The XLogP/MW chemical space used to define the AD in this study did however demonstrate relevance in characterising and further investigating the performance of the QSAR models over chemical space, despite using physicochemical descriptors which were considerably different from the SMILES-based descriptors originally used to build the feature space of the QSAR models. From this, it was found that particular regions of the chemical space were significantly more problematic for the QSAR models than others, despite in one case being a data-dense region with an approximately equal statistical distribution of both classes; this particular region significantly contributed to lowering the most optimal QSAR model overall accuracy scores from within range of ~80% to within range of ~70%. It was also further uncovered that, despite underperforming in terms of the majority of summary graphs and metrics, isomap outperformed over certain regions of chemical space that other techniques were negatively impacted by; indeed each technique carried a unique insight into navigating toxicological space, although the exponential distribution of the dataset in chemical space meant that higher performance over the most central and densely populated regions carried the largest weight for determining overall performance.

Future expansions to this study could entail a wider use of other advanced dimensionality reduction techniques, as well as different (and perhaps more balanced) datasets for other toxicological endpoints for comparison, especially with application to datasets which do not satisfy the *N ≤ D* + 1 condition of Cover’s theorem [12]. Other hyperparameter optimisation methods may also be explored and utilised, such as random searches and Bayesian optimisation (or perhaps even separate DNN models for predicting optimal hyperparameter values) [26]. The AD could in future be defined through a more advanced means, such as density-based methods [27], while also deeper investigations into the properties of problematic regions of chemical space (as well as adjustment of QSAR models to detect and navigate such spaces) could offer further innovation for this field.

## Figures and Tables

**Figure A1:**
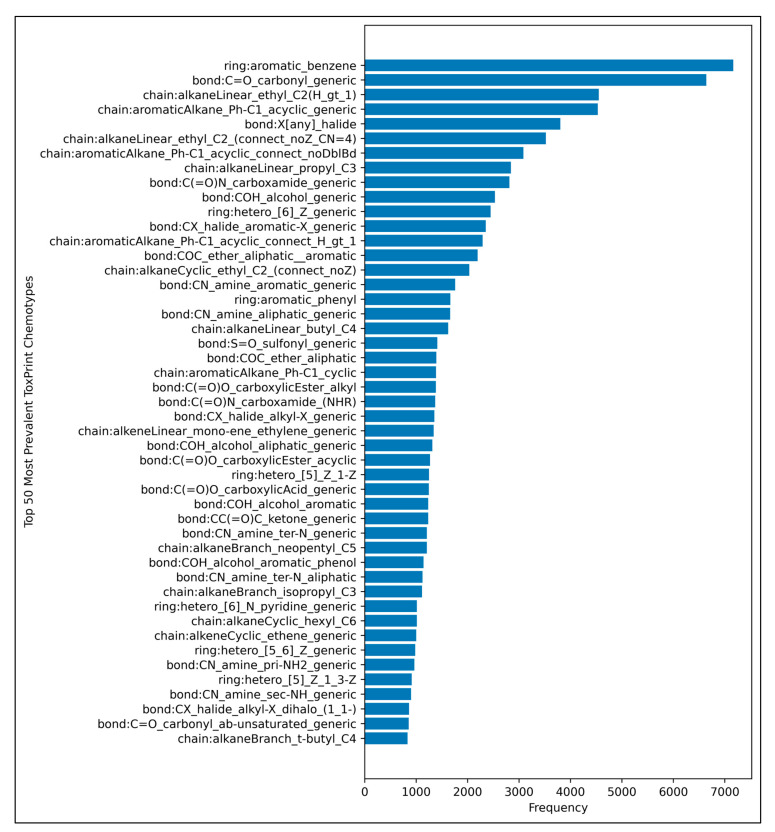
Bar chart of frequencies of the top 50 most abundant ToxPrint chemotypes, for the curated 11,268 molecules used in the study, using the ToxPrint ChemoTyper software tool [28].

**Figure A2:**
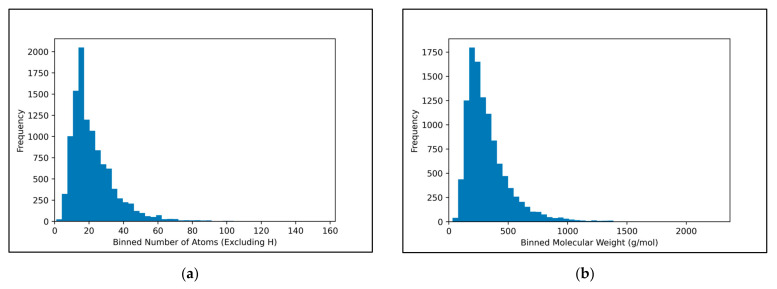
Histograms characterising the distribution of the 11,268 curated molecules in terms of: (**a**) Number of atoms (excluding H atoms); (**b**) Molecular weight (g/mol), according to PubChem [13]; Note that in both cases, 50 bins were used.

**Figure A3:**
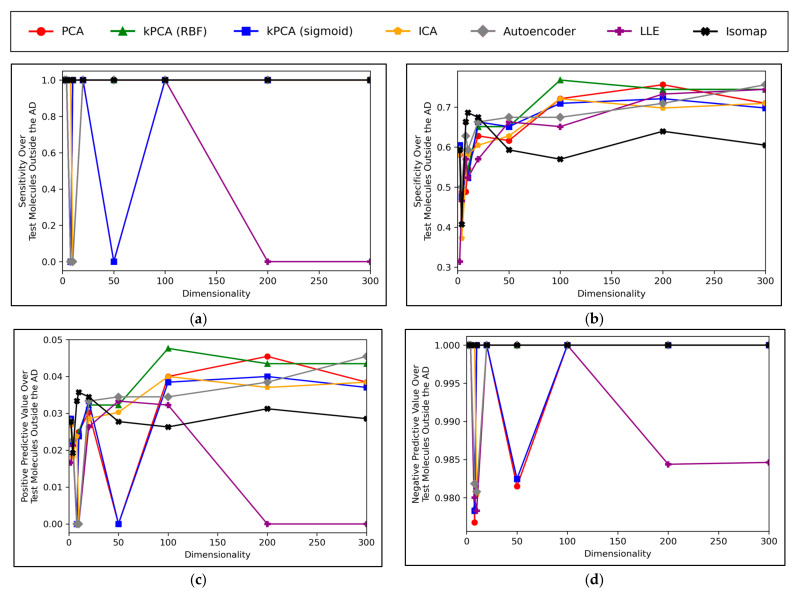
Comparative performance metrics graphs for all optimised dimensionality reduction techniques, over ascending dimensionalities, for molecules outside of the defined AD, in terms of: (**a**) Sensitivity (**b**) Specificity; (**c**) PPV (positive predictive value); (**d**) NPV (negative predictive value).

**Figure A4:**
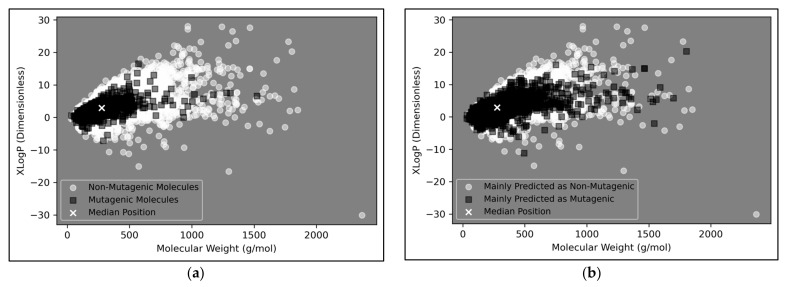
Alternatively coloured version of Figure 9, for the perusal of colourblind readers; Visualisations of the distributions in chemical space of; (**a**) Molecules in the dataset that were mutagenic versus non-mutagenic; (**b**) Molecules in the dataset that were mainly predicted as mutagenic versus mainly predicted as non-mutagenic (according to the autoencoder powered QSAR model at 300 dimensions, across all iterations); Note that in all cases, median position of the entire dataset is marked, for reference.

## Data Availability

Data from the 2014 Ames/QSAR International Challenge Project is unavailable, due to legal restrictions.
